# Investigations of segmental arterial stiffness in a cross‐sectional study on young adult male trained swimmers, cyclists, and non‐trained men

**DOI:** 10.14814/phy2.70186

**Published:** 2025-01-01

**Authors:** Masato Nishiwaki, Daisuke Kume, Naoyuki Matsumoto

**Affiliations:** ^1^ Faculty of Engineering Osaka Institute of Technology Osaka Japan; ^2^ Faculty of Information Science and Technology Osaka Institute of Technology Hirakata, Osaka Japan; ^3^ Faculty of Environmental Symbiotic Sciences Prefectural University of Kumamoto Kumamoto Japan

**Keywords:** aerobic exercise, arteriosclerosis, vascular function

## Abstract

Contrary to cardiovascular risk reductions by aerobic exercise, arterial stiffness, as assessed by brachial‐ankle pulse wave velocity (PWV), is higher in swimmers and controls than in other aerobically trained individuals. The main muscles actively recruited in swimming are in the upper limbs, so this study aimed to investigate heart‐brachial PWV in swimmers and to compare arterial stiffness indices between modes and measurement localities. Subjects comprised 60 individuals (18–22 years), including 20 untrained controls (Con), 20 aerobically trained cyclists (Aero), and 20 swimmers (Swim). Characteristics and strength did not differ, but peak oxygen uptake was significantly higher in Aero and Swim than in Con. Brachial‐ankle PWV was significantly lower in Aero than in Con and Swim and no significant difference was observed between Con and Swim (Con, 1070 ± 115; Aero, 916 ± 109; Swim, 1035 ± 91 cm/s). Nevertheless, heart‐brachial PWV was significantly lower in Swim than in Con and tended to be lower in Swim than in Aero (Con, 344 ± 25; Aero, 330 ± 41; Swim, 308 ± 31 cm/s). Heart‐ankle PWV was significantly lower in both Swim and Aero than in Con (Con, 618 ± 47; Aero, 580 ± 54; Swim, 576 ± 43 cm/s). Therefore, these findings indicate that swimmers can develop segment‐specific reductions in heart‐brachial arterial stiffness, unlike aerobically trained cyclists.

## 1 INTRODUCTION

Pulse wave velocity (PWV) is widely established as a standard index of arterial stiffness (Laurent et al., 2006), and arterial stiffening is known to occur with aging (Avolio et al., 1985; Tomiyama et al., 2003). Large arteries (aortic) or conduit arteries play a central role in cushioning (buffering) functions, and the functions contribute to marked increases in systemic blood flow during aerobic exercise (Tanaka, 2020). The carotid‐femoral PWV (cfPWV) has been identified as the reference standard for aortic arterial stiffness (Laurent et al., 2006). Further, brachial‐ankle PWV (baPWV) has been generally utilized as a traditional standard index of arterial stiffness, which reflects the stiffness of the abdominal aorta and the leg arteries (Sugawara et al., 2005; Sugawara & Tanaka, 2015). Although systemic parameters (i.e., heart‐ankle PWV; haPWV) have come to be widely used, heart‐brachial PWV (hbPWV) has been recently proposed as a novel index of arterial stiffness for the upper limbs (Ogawa et al., 2020; Sugawara et al., 2019). Various parameters are used, but arterial stiffness is significantly related to not only cardiovascular disease risks (Vlachopoulos et al., 2010), but also maximal oxygen uptake (Tomoto et al., 2017; Vaitkevicius et al., 1993). Indeed, cfPWV or baPWV is lower in regular aerobically trained participants than in sedentary participants (Nishiwaki, Takahara, & Matsumoto, 2017; Otsuki et al., 2007). Aerobic exercise training can also reduce PWV parameters even in young participants (Kakiyama et al., 2005). Therefore, regular aerobic exercise is closely associated with adaptations of arterial stiffness.

Swimming is one of the most representative modes of aerobic exercise and is widely promoted and recommended by many organizations (American College of Sports Medicine Position Stand., 1998; Fletcher et al., 1992; World Hypertension League, 1991). Nevertheless, PWV‐assessed arterial stiffness in swimmers does not differ significantly from that in sedentary controls and is significantly higher than that in runners or cyclists (Nishiwaki, Takahara, & Matsumoto, 2017; Nualnim et al., 2011). The physiological reasons why PWV‐assessed arterial stiffness statuses in swimmers differ from those in other aerobically trained individuals and do not differ from those in sedentary controls remains unclear.

Swimming is generally marked by prominent differences from land‐based aerobic exercises, particularly in terms of the main muscle segments actively recruited (i.e., upper limbs vs. lower limbs) (Aspenes & Karlsen, 2012). Front‐crawl swimming is the most commonly used swimming style, in which leg kicking contributes to approximately ~10% of the velocity and constitutes approximately 5%–25% of the swimmer's energy output (Morris et al., 2016, 2017; Ogita et al., 1996). In more recent evidence, although leg kicking is important to maintaining posture, kicking during high‐speed swimming is also known to become a factor in resistance (Narita et al., 2018). The arm stroke is thus generally accepted to be responsible for most of the velocity or energy output during swimming and plays the role of the main muscles actively recruited (Huang et al., 2010). However, previous studies of arterial stiffness in swimmers have utilized cfPWV or baPWV and have not evaluated PWV of the upper limbs (Cheung et al., 2021; Nishiwaki, Takahara, & Matsumoto, 2017; Nualnim et al., 2011). This discrepancy between the main muscles actively recruited and the main segments evaluated may thus contribute to the assessment results of arterial stiffness in swimmers. We therefore considered that hbPWV measurements would be important to unravel the status of arterial stiffness in swimmers. Such investigations would contribute to a better understanding and bridging of the knowledge gap for the physiological mechanisms of aerobic exercise‐induced reduction in arterial stiffness (i.e., local or systemic factors). To the best of our knowledge, however, no data are available regarding hbPWV in swimmers.

One‐legged exercises modulate arterial stiffness only in the exercised leg, and not in the control leg, and the effects of exercise on arterial stiffness thus appear to be strongly influenced by local factors, regardless of the mode of exercise (Heffernan et al., 2006; Oda et al., 2024; Sugawara et al., 2003; Yamato et al., 2017). We therefore hypothesized that hbPWV would be lower in swimmers whose main trained limbs are the arms than in control individuals and other aerobically trained individuals, including cyclists, whose main trained limbs are the legs. The present study thus aimed to test these hypotheses using a cross‐sectional study design and to clarify the differences in arterial stiffness between exercise types and measurement localities.

## 2 METHODS

### 2.1 Participants

The appropriate sample size was first determined by power analysis using G*Power version 3.1 (Dusseldorf, Germany). A total sample size of 60 (20 participants per group) was needed to detect an effect size (ES) (f) of 0.42 based on previous literature on arterial stiffness (Nishiwaki, Takahara, & Matsumoto, 2017) at 80% power with an *α* value of 5% using one‐way analysis of variance (ANOVA). We therefore planned to recruit 20 participants per group (total sample size, 60 participants) in this study.

Sixty Japanese male college students (age range, 18–22 years) responded to local advertisements and referrals to participate in the present study. These students comprised 20 untrained individuals as an age‐matched control group (Con group), 20 competitive cyclists (aerobically trained athletes; Aero group), and 20 competitive swimmers (Swim group). Competitive sports career lengths in the Aero and Swim groups were > at least 2 years. On average, the cyclists and swimmers had been exercising 4–5 times/week. Cyclists regularly cycled approximately between 250 km/week and 300 km/week on the load, and the cyclists usually participated in 50‐, 100‐, and 150‐km races. Swimmers regularly swam approximately between 28,000 m/week and 35,000 m/week, mainly using front‐crawl swimming and the swimmers usually participated in 50‐, 100‐, 200‐, and 400‐m races. No cyclists or swimmers had any history of participation in regular high‐intensity resistance training, which might increase arterial stiffness in young subjects (Miyachi, 2013). Con group participants had not engaged in any regular exercise programs, such as through participation in clubs, teams, or extracurricular sporting activities for at least the previous 2 years. All participants were nonsmokers and were not presently taking any medications or supplements. None had any history of chronic diseases that could affect cardiovascular and metabolic health or their ability to exercise. The purposes, procedures, and risks of the study were explained to each participant. Written, informed consent was obtained from each participant before enrolment in the study, which was reviewed and approved by the Human Ethics Committee at the Osaka Institute of Technology (approval no; 2022‐09) and developed in accordance with the guidelines of the Declaration of Helsinki.

### 2.2 Experimental procedures

All measurements were conducted in a quiet, air‐conditioned room at 22–24°C. Participants abstained from vigorous exercise for at least 24 h and from caffeine, alcohol, and food for ≥4 h before testing, to avoid acute effects on arterial stiffness. Compliance was confirmed using a checklist questionnaire and face‐to‐face interview. All of the participants arrived at the laboratory and rested for at least 30 min, then body composition, hemodynamic parameters, arterial stiffness, handgrip strength, and peak oxygen uptake were assessed.

### 2.3 Body composition

According to previous studies (Nishiwaki et al., 2014; Nishiwaki, Nakashima, et al., 2017), height without footwear and body composition (weight, lean body mass (LBM), and body fat) in light clothing were determined using a bioelectrical impedance analysis (TBF‐410, Tanita Co, Tokyo, Japan). BMI was calculated as body weight (kg) divided by the height square (m).

### 2.4 Hemodynamic parameters and arterial stiffness

After resting for ≥15 min in a supine position, PWV, BP, and heart rate (HR) were measured using an automated VS‐1500AE/AN device (Fukuda Denshi, Tokyo, Japan), as described (Kume et al., 2021; Nishiwaki et al., 2019). Electrodes for electrocardiography (ECG) were placed on both wrists, and a heart sound microphone was placed at the left edge of the sternum. Lead 1 ECG and heart sounds were measured simultaneously with BP and PWV. Cuffs to measure BP and PWV were wrapped around both upper arms and ankles, then hbPWV, baPWV, and heart‐ankle PWV (haPWV), reflecting systemic arterial stiffness between heart and ankle, were used as indices of arterial stiffness. Each PWV was calculated by dividing the distance between the two arterial recording sites by the transit time. Path lengths were calculated according to the height of the participant (Kume et al., 2021; Nishiwaki et al., 2019). Briefly, vascular lengths between the suprasternal notch and the brachium (L_b_ = 0.2195 × height (in cm) − 2.0734) and between the suprasternal notch and the ankle (L_a_ = 0.8129 × height (in cm) + 12.328) were calculated, respectively (Yamashina et al., 2002). The values of L_b_ and the transit time between the second heart sound and the upper arm (T_b_) were utilized to calculate hbPWV. The values of L_a_ − L_b_ and the transit time between the upper arm and the ankle (T_ba_) were utilized to calculate baPWV. Finally, the formula for calculating the distance between the heart valve and the ankle (L) was not officially disclosed, and the values were automatically calculated by the measurement device (However, the following formula may be used: L = 0.77685 × height (in cm) − 1.7536, manufacturer's informal information). The L values and the total transit time of T_b_ + T_ba_ were utilized to calculate haPWV. Ankle‐brachial index (ABI) was also obtained by dividing ankle systolic BP by brachial systolic BP. Day‐to‐day coefficients of variation (CVs) for hbPWV, baPWV, and haPWV measurements on two separate days (i.e., reproducibility) were all <5%, as described elsewhere (Nishiwaki et al., 2020; Ogawa et al., 2020).

### 2.5 Handgrip strength

Handgrip strength was measured using a dynamometer (T.K.K. 5001 Grip‐A; Takeikiki, Niigata, Japan) according to the methods of previous studies (Nishiwaki et al., 2014, 2015). The width of the handle was adjusted to fit each hand, and the elbow was fully extended to measure maximal strength twice, then the highest values were reported for the stronger hand. We further expressed handgrip strength data normalized to body weight, as follows: handgrip strength (*N*/kg) = measured value × 9.8/body weight. The constant 9.8 represents the factor for converting from kilograms to Newtons. Day‐to‐day CV for handgrip strength was 4.1 ± 0.8%.

### 2.6 Peak oxygen uptake

Peak oxygen uptake (VO_2_peak) was determined during incremental cycle ergometer exercise (75XL III; Konami, Tokyo, Japan) according to the methods of previous study (Nishiwaki, Takahara, & Matsumoto, 2017). Pedaling rate was kept constant at 60 rpm for all participants. Exercise intensity was first set at 60 W, then increased by 30 W every 2 min until exhaustion. Exhaustion was defined by the participant being unable to maintain the pedaling rate. In this study, respiratory gases were collected and analyzed every 15 s using an automatic gas analyzer with a mixing chamber (AR10; Arco System, Chiba, Japan) that was calibrated and confirmed before each test according to the instructions from the manufacturer (Nishiwaki et al., 2019). Collected data were averaged every 30 s and VO_2_peak was determined as the highest value during incremental exercise. Day‐to‐day CV for VO_2_peak was 5.3 ± 1.5%.

### 2.7 Statistics

Results are presented as mean ± standard deviation. The normal distribution and homoscedasticity of all data were confirmed using the Kolmogorov–Smirnov and Levene's tests. Each parameter was compared among the three groups using one‐way ANOVA and analysis of covariance (ANCOVA) that included BMI and mean BP as covariates. We selected these covariates based on previous literature suggesting strong associations with arterial stiffness (Benetos et al., 1993; Nishiwaki et al., 2014; Nishiwaki, Takahara, & Matsumoto, 2017; Ogawa et al., 2020; Vaitkevicius et al., 1993; Yamamoto et al., 2009). In the case of significant *F* values, Bonferroni correction was used for post hoc multiple comparisons. All data were statistically analyzed using IBM SPSS Statistics 29 (IBM Japan, Tokyo, Japan). Graphical representation was performed using GraphPad Prism version 9.2.0 (GraphPad Software by Dotmatics, Boston, USA). To quantify the magnitude of differences between the two groups, ES (d) was calculated using G*Power 3.1. Differences were considered significant at the level of *p* < 0.05.

## 3 RESULTS

Table 1 shows the physical characteristics and hemodynamic parameters of participants in all groups. No significant differences were observed in age, height, weight, BMI, or body fat. LBM was significantly higher in the Swim group than in the Con group. Brachial SBP was slightly higher and brachial DBP was significantly lower in the Swim group than in the Con and Aero groups. As a result, brachial PP was significantly higher in the Swim group than in the Con and Aero groups. Ankle DBP was also significantly lower in the Swim group than in the Con and Aero groups. However, besides these differences, no significant differences in HR, BPs, or ABI were observed among groups. Further, no significant differences in handgrip strength were found in terms of either absolute or relative values per body weight among the three groups. VO_2_peak was significantly higher in both the Aero and Swim groups than in the Con group for both absolute and relative values per body weight, but no significant difference was evident between Aero and Swim groups.

**TABLE 1:** Physical characteristics and hemodynamic parameters of study participants.

Parameters	Con	Aero	Swim	One‐way ANOVA	
Number of participants	*n* = 20	*n* = 20	*n* = 20	–	–
Age, y	21 ± 1	20 ± 1	20 ± 1	*F* = 3.126	*p* = 0.051
Height, cm	169.4 ± 5.9	168.8 ± 5.6	171.8 ± 4.3	*F* = 1.791	*p* = 0.176
Weight, kg	62.1 ± 8.5	63.3 ± 9.1	66.0 ± 9.8	*F* = 0.940	*p* = 0.397
Body mass index, kg/m^2^	21.7 ± 2.6	22.2 ± 2.8	22.4 ± 3.7	*F* = 0.313	*p* = 0.733
Body fat, %	18.9 ± 4.4	20.1 ± 5.6	18.2 ± 6.5	*F* = 0.530	*p* = 0.591
Lean body mass, kg	48.2 ± 9.1	50.2 ± 4.5	53.6 ± 4.8^a^	*F* = 3.451	*p* = 0.039
Heart rate, beats/min	65 ± 9	63 ± 7	64 ± 13	*F* = 0.184	*p* = 0.832
Brachial systolic BP, mmHg	125 ± 8	126 ± 10	128 ± 9	*F* = 0.538	*p* = 0.587
Brachial diastolic BP, mmHg	72 ± 6	74 ± 7	69 ± 6^c^	*F* = 3.676	*p* = 0.032
Brachial mean BP, mmHg	91 ± 6	92 ± 7	89 ± 7	*F* = 1.018	*p* = 0.368
Brachial PP, mmHg	53 ± 8	52 ± 7	59 ± 7^a^, ^c^	*F* = 6.301	*p* = 0.003
Ankle systolic BP, mmHg	139 ± 10	141 ± 15	137 ± 9	*F* = 0.728	*p* = 0.487
Ankle diastolic BP, mmHg	70 ± 6	69 ± 8	64 ± 6^a^	*F* = 4.029	*p* = 0.023
Ankle mean BP, mmHg	94 ± 6	92 ± 11	89 ± 8	*F* = 1.479	*p* = 0.236
Ankle PP, mmHg	70 ± 10	72 ± 11	73 ± 7	*F* = 0.646	*p* = 0.528
ABI, unit	1.09 ± 0.07	1.01 ± 0.08	1.05 ± 0.07	*F* = 2.503	*p* = 0.091
Handgrip strength, kg	39.6 ± 4.6	43.5 ± 6.0	41.7 ± 5.0	*F* = 2.223	*p* = 0.119
Handgrip strength, N/kg	6.30 ± 1.11	6.92 ± 0.91	6.24 ± 0.82	*F* = 2.740	*p* = 0.074
Peak oxygen uptake, L/min	2.55 ± 0.61	3.42 ± 0.51^b^	3.30 ± 0.39^b^	*F* = 16.809	*p* < 0.001
Peak oxygen uptake, mL/kg/min	41.0 ± 8.5	54.9 ± 8.5^b^	50.4 ± 6.1^b^	*F* = 16.356	*p* < 0.001

*Note*: Data are expressed as mean ± SD.

Abbreviations: ABI, ankle‐brachial index; Aero, competitive cyclist group (i.e., aerobic‐trained group); BP, blood pressure; Con, untrained age‐matched control group; PP, pulse pressure, Swim, competitive swimmer group.

^a^ *p* < 0.05 versus Con.

^b^ *p* < 0.01 versus Con.

^c^ *p* < 0.05 versus Aero.

**FIGURE 1:**
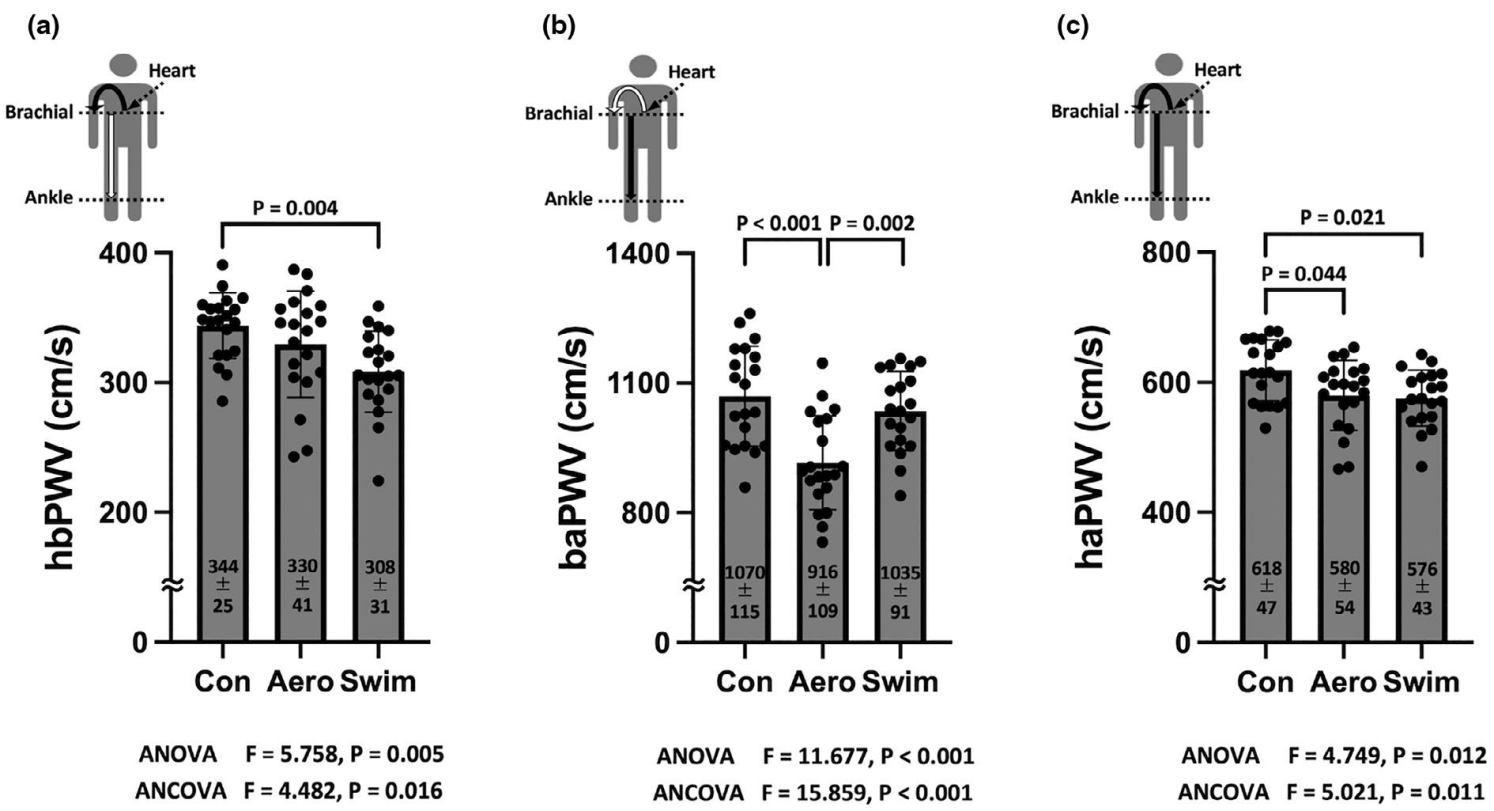
Comparisons of hbPWV (a), baPWV (b), and haPWV (c) in Con, Aero, and Swim groups. (a) hbPWV, heart‐brachial pulse wave velocity reflecting arterial stiffness in the upper limbs from the aorta to the brachium (black arrow in the illustrations at the top left); (b) baPWV, brachial‐ankle pulse wave velocity reflecting both central and lower limbs arterial stiffness from the brachium (i.e., corresponding to an unspecified point in the thoracoabdominal region) to the ankle (black arrow in the illustrations at the top left); (c) heart‐ankle PWV, heart‐ankle pulse wave velocity reflecting systemic arterial stiffness (black arrows in the illustrations at the top left); Con, untrained, age‐matched, control individuals; Aero, competitive cyclists (i.e., aerobically trained athletes); Swim, competitive swimmers; ANOVA, analysis of variance; ANCOVA, analysis of covariance. Black arrows indicate measurement parts and white arrows indicate non‐measurement parts in the illustrations at the top left, respectively. Small, filled circles in the bar graph indicate individual values. Data are expressed as mean ± SD.

Figure 1 shows segmental arterial stiffness in all groups. Interestingly, ANOVA indicated that hbPWV was significantly lower in the Swim group than in the Con group (ES = 1.28), whereas no significant difference in hbPWV was found between the Con and Aero groups (ES = 0.41). The value of hbPWV was not significantly lower in the Swim group than in the Aero group after Bonferroni corrections (*p* = 0.145; ES = 0.61). On the contrary, baPWV was significantly lower in the Aero group than in the Con (ES = 1.37) or Swim groups (ES = 1.19), whereas no significant difference in baPWV was found between Con and Swim groups (ES = 0.34). Finally, in terms of the systemic index of arterial stiffness, haPWV was significantly lower in the Aero (ES = 0.76) and Swim (ES = 0.93) groups than in the Con group, whereas no significant difference in haPWV was found between the Aero and Swim groups (ES = 0.08). These results remained unchanged after normalizing hbPWV, baPWV, or haPWV to BP and physical fitness‐related parameters, due to strong associations with arterial stiffness, when analyzed by ANCOVA.

## 4 DISCUSSION

The major new findings were that hbPWV was significantly lower in swimmers, but not in aerobically trained cyclists, than in control individuals. This suggests that swimmers have a segment‐specific lower heart‐brachial arterial stiffness and that regular aerobic training, including swimming, strongly induces localized arterial stiffness adaptations.

To discuss vascular adaptations to swimming exercise, the present study focused on the discrepancy between the main muscles actively recruited in swimming and the arterial segments evaluated for arterial stiffness. Recent studies have shown that hbPWV reflects arterial stiffness of the upper limbs from the heart to the brachium (Sugawara et al., 2019). This variable may serve as a novel marker of arterial stiffness of the proximal aorta, and the reproducibility and validity of measurement are high (Sugawara et al., 2019). Our findings therefore suggest that heart‐brachial arterial stiffness was significantly lower in swimmers, but not in aerobically trained cyclists, than in control individuals, in turn suggesting that swimmers show segment‐specific lower heart‐brachial arterial stiffness, unlike aerobically trained cyclists. Regular swimming exercise per se may thus reduce upper arterial stiffness for the regions of muscles actively recruited in swimming and changes in arterial stiffness with exercise may be strongly influenced by exercise‐induced local factors.

We can only speculate on the physiological mechanisms underlying the results obtained in the present study. First, vascular responses and adaptations to local exercise or local stimulations have been previously found only in the exercised or experimental parts (Green et al., 2017; Heffernan et al., 2006; Miyachi et al., 2001; Soares et al., 2018; Sugawara et al., 2003; Yamato et al., 2017). These results imply that local factors are more important than systemic factors, for reductions in arterial stiffness or vascular adaptations. Repetitive increases in blood flow and shear stress with exercise in the active limbs may thus enhance nitric oxide bioavailability in the vascular endothelium, thereby reducing localized arterial stiffness (Green et al., 2017; Laughlin et al., 2008). Because the major active limbs during exercise differ between swimming (i.e., upper limbs) and cycling (i.e., lower limbs) (Alkatan et al., 2016), swimmers can achieve a segment‐specific lower heart‐brachial arterial stiffness, unlike aerobically trained cyclists. Accordingly, swimming‐related arm exercises may result in localized alternation in upper arm arterial stiffness.

Our results also show significant differences in baPWV between Aero and CON, but not between Swim and CON. Previously, arterial stiffness in swimmers did not differ from that of controls and was significantly higher than that of other aerobically trained individuals (Nishiwaki, Takahara, & Matsumoto, 2017; Nualnim et al., 2011). Our findings for baPWV are consistent with the previous results. However, it should be considered to discuss the measurement principle for baPWV. In particular, the pulse wave does not travel directly from the brachial arteries to the posterior tibial arteries in the same arterial tree (Sugawara & Tanaka, 2015), and the baPWV transit time is determined from the temporal delay between the brachial (i.e., corresponding to an unspecified point in the thoracoabdominal region) and ankle waveforms (Sugawara et al., 2005; Sugawara & Tanaka, 2015). In the case of a compliant artery only in the heart‐brachial segment, such as swimmers, the slower pulse wave arrives at the brachial segment than other body segments. However, at the thoracoabdominal level, the actual faster pulse wave arrives and passes through the ankle. The discrepancy between the slower pulse wave arrival of the heart‐brachial segment and the actual normal or faster pulse wave arrival at the thoracoabdominal level would reduce the time interval (temporal delay) between the brachial and ankle evaluation sites. The shorter time interval than the actual physiological status can thus affect higher baPWV values. Therefore, our findings for baPWV suggest that the index cannot be used to assess arterial stiffness of individuals with a compliant artery only in the heart‐brachial segment such as swimmers.

Interestingly, although our data show differing results for hbPWV and baPWV among Swim and Aero, a systemic parameter of arterial stiffness (i.e., haPWV) was significantly lower in both Swim and Aero than in Con. Since haPWV is an index of systemic arterial stiffness from the root of the aorta to the posterior tibial arteries (Ogawa et al., 2020; Tomoto et al., 2017), individuals performing aerobic exercise, including swimmers, can be interpreted as achieving overall lower systemic arterial stiffness, as supported by previous interventional studies (Alkatan et al., 2016; Nualnim et al., 2012; Yuan et al., 2016). Previous and our results showed baPWV of the swimmers is not lower than that of the control groups. However, some cross‐sectional studies have reported higher compliant artery or lower arterial stiffness in swimmers using different indices of carotid artery compliance and cardio‐ankle vascular index (CAVI) in addition to hbPWV (Nishiwaki, Takahara, & Matsumoto, 2017; Nualnim et al., 2011). All things considered, our results indicate that the swimmers as well as runners and cyclists can have an overall lower arterial stiffness of the systemic segment despite significant segment‐specific effects.

Regular swimming might eventually be related to lower cardiovascular disease risk or mortalities. Some large cohort studies have demonstrated significant reductions in cardiovascular disease or all‐cause mortality were associated with participation in swimming as well as other exercises (Chase et al., 2008; Oja et al., 2017). A recent study has shown that hbPWV undergoes a linear augmentation with age, commencing from an early adult life stage onward, rendering it a potential marker for discerning cardiovascular risk (Sugawara et al., 2024). Our cross‐sectional results indicate that swimmers have lower arterial stiffness of the systemic segment despite significant segment‐specific effects. Regular swimming might thus reduce risks of cardiovascular disease and all‐cause mortality. However, further robust evidence is required to clear these points.

One potential limitation of the present study was that the cross‐sectional study design with a comparatively small sample size limited the ability to determine a cause‐and‐effect relationship regarding the effects of swimming exercise. Further interventional studies are warranted to elucidate whether regular swimming exercise actually induces reductions in segment‐specific arterial stiffness.

## 5 CONCLUSION

Our results indicate that hbPWV is significantly lower in Swim, but not in Aero, than in Con. Therefore, although swimmers overall can show lower arterial stiffness of the systemic segment, these findings suggest that swimmers develop segment‐specific reductions in heart‐brachial arterial stiffness, unlike aerobically trained cyclists. Our findings also raise the possibility that aerobic exercise, including swimming, strongly induces localized arterial stiffness adaptations (i.e., the importance of local factor effects).

## AUTHOR CONTRIBUTIONS

MN conceived and designed the study, performed the study, analyzed the data, interpreted the data, and drafted the manuscript; DK and NM interpreted the data. All authors read and approved the final version of the article.

## FUNDING INFORMATION

This study was supported in part by JSPS KAKENHI to MN (24K02837) and to DK (23K10649).

## CONFLICT OF INTEREST STATEMENT

The authors declare that there is no conflict of interest regarding the publication of this article.

## ETHICS APPROVAL AND PATIENT CONSENT STATEMENT

The purposes, procedures, and risks of the study were explained to each participant. Written, informed consent was obtained from each participant before enrolment in the study, which was reviewed and approved by the Human Ethics Committee at the Osaka Institute of Technology (approval no; 2022‐09) and developed in accordance with the guidelines of the Declaration of Helsinki.

## Data Availability

The raw data supporting the conclusions of this article will be made available by the authors, without undue reservation. Because Ethics Committee of our institution restricted for participant privacy protection, our raw data cannot be shared publicly via on‐line network.
